# Genome Sequencing of Four Multidrug-Resistant *Enterobacter aerogenes* Isolates from Hospitalized Patients in Brazil

**DOI:** 10.3389/fmicb.2016.01649

**Published:** 2016-10-27

**Authors:** Ana Laura Grazziotin, Newton M. Vidal, Jussara K. Palmeiro, Libera Maria Dalla-Costa, Thiago M. Venancio

**Affiliations:** ^1^Laboratório de Química e Função de Proteínas e Peptídeos, Centro de Biociências e Biotecnologia, Universidade Estadual do Norte Fluminense Darcy RibeiroCampos dos Goytacazes, Brazil; ^2^National Center for Biotechnology Information, National Library of Medicine, National Institutes of HealthBethesda, MD, USA; ^3^Laboratório de Bacteriologia, Unidade Laboratório de Análises Clínicas, Hospital de Clínicas, Universidade Federal do ParanáCuritiba, Brazil; ^4^Faculdades e Instituto de Pesquisa Pelé Pequeno PríncipeCuritiba, Brazil

**Keywords:** Brazil, *Enterobacter aerogenes*, genome sequencing, multidrug resistance, carbapenem resistance

## Background

*Enterobacter aerogenes* is a motile, non-spore forming, Gram-negative bacteria from the *Enterobacteriaceae* family. *Enterobacter* spp. have emerged as multidrug-resistant (MDR) nosocomial bacteria, especially in intensive care units (Loiwal et al., 1999; Piagnerelli et al., 2002). Therefore, over the last decade *Enterobacter* spp. were included in the ESKAPE group, which also comprises *Enterococcus faecium, Staphylococcus aureus, Klebsiella pneumoniae, Acinetobacter baumannii*, and *Pseudomonas aeruginosa* (Rice, 2008; Boucher et al., 2009). Further, bloodstream infections with MDR *E. aerogenes* have been associated with high mortality rates (Davin-Regli and Pagés, 2015).

Hospital outbreaks due to *E. aerogenes* have been reported in Europe since the mid-1990s and have been related to an epidemic extended-spectrum beta-lactamase (ESBL) clone carrying the *bla*_TEM-24_ gene (Bosi et al., 1999; Galdbart et al., 2000; Dumarche et al., 2002; Salso et al., 2003). Constitutive AmpC a (beta-lactamase) overexpression is the major cephalosporin resistance mechanism in *Enterobacter* spp., happening more often than the acquisition of *amp*C genes through the activity of mobile genetic elements (Perez-Perez and Hanson, 2002). Further, the increased expression of ESBLs led to the adoption of carbapenems to treat *E. aerogenes* infections (Perez-Perez and Hanson, 2002; Davin-Regli and Pagés, 2015).

Carbapenems have been considered the antibiotic of choice for treating patients infected with ESBL-producing *Enterobacteriaceae* (Vardakas et al., 2012). However, emergence of carbapenem-resistant *E. aerogenes* isolates during carbapenem therapy of hospitalized patients (Chen et al., 2008), cases of sepsis due to carbapenem-resistant *E. aerogenes* after liver transplantation (Chen et al., 2009) and hospital disseminations of carbapenemase-producing *E. aerogenes* have been recently reported in several countries (Lavigne et al., 2013; Kuai et al., 2014; Qin et al., 2014; Pulcrano et al., 2016). Acquisition and expression of carbapenemases constitute the primary mechanism underlying the development of carbapenem resistance (Rapp and Urban, 2012). Nevertheless, loss of function mutations in porin genes and increased expression of efflux pumps or their regulators have also been associated with carbapenem resistance profiles (Pradel and Pages, 2002; Yigit et al., 2002; Bornet et al., 2003).

Broad-spectrum antimicrobial-resistant *E. aerogenes* isolates, some resistant to carbapenems (Qin et al., 2014) and last-line therapeutic options such as colistin (Diene et al., 2013), have been responsible for outbreaks in the United States of America (Wong et al., 2010), China (Qin et al., 2014), Japan (Goshi et al., 2002), France (Diene et al., 2013), Fiji (Narayan et al., 2009) and Brazil (Tuon et al., 2015). However, few reports related to *E. aerogenes* epidemiology, pathogenesis, and molecular characterization have been conducted in Brazil. Recently, five panresistant *E. aerogenes* isolates were reported in a Brazilian teaching hospital, resulting in a high mortality rate (37.5%) among 16 infected patients (Tuon et al., 2015). We have observed high prevalence (>20%) of ESBL-producing *Enterobacteriaceae* spp., in particular *K. pneumoniae* and *E. aerogenes*, in our hospital since 2003 (Nogueira Kda et al., 2014, 2015). Previous molecular characterization studies conducted over 5 years in our hospital showed high prevalence of *bla*_CTX-M2_, _-M15_, _-M59_, *bla*_SHV-2_ and *bla*_TEM_ genes in *Enterobacter* spp. isolates (Nogueira Kda et al., 2014, 2015). The presence of *bla*_PER-2_ was also detected in a few isolates (Nogueira Kda et al., 2014, 2015). Given the severity of *E. aerogenes* infections and the urgent need to better understand the genetic basis of multidrug resistance, here we report the whole-genome sequencing and resistance gene repertoire of four multidrug-resistant *E. aerogenes* isolated from hospitalized patients in Brazil.

## Methods

### Sample collection and identification

*E. aerogenes* isolates C10, D2, D3, and E9 were obtained between 2006 and 2012 from patients hospitalized in wards or intensive care units at the Hospital de Clínicas of the Universidade Federal do Paraná (Curitiba, Brazil). The main selection criterion for genome sequencing was the MDR phenotype, particularly in carbapenem resistant isolates. The negative laboratory tests for carbapenemases were also taken into account, as divergent enzymes or alternative resistance mechanisms could be relevant to the observed MDR phenotypes. C10 and D2 samples were isolated from different body sites of the same patient. Isolates were grown in selective medium with an ertapenem disk (10 ug) and stored at −80°C in trypticase soy broth containing glycerol 15%. Identification of isolates was performed using Vitek® 2 Compact (BioMérieux S.A., Marcy l'Etoile, France) and by mass spectrometry using Microflex LT instrument (Bruker Daltonics, Bremen, Germany). This study was carried out in accordance with the Brazilian legislation and was approved by the Institutional Ethics Review Board of the Hospital de Clínicas, Universidade Federal do Paraná (IRB#: 2656.263/2011-11). Our study involved only bacterial isolates and no human specimens were analyzed or stored. Further, we used no patient information other than the anatomical sites from where the isolates were collected. Therefore, the same Ethics Review Board exempted us from obtaining informed consent forms.

### Resistance profile analysis

#### Antimicrobial susceptibility testing

Isolates were tested by agar dilution against 15 antibiotics according to the Clinical and Laboratory Standard Institute guidelines (CLSI, 2015a). Minimal inhibitory concentration (MIC) was interpreted as recommended by CLSI standards (CLSI, 2015b). Polymyxin, tigecycline and fosfomycin breakpoints were interpreted using EUCAST standards (Eucast, 2016). Modified Hodge test (MHT), double-disk synergy and hydrolysis assay were performed to determine the carbapenem resistance phenotypes, as previously described (Carvalhaes et al., 2010; Eucast, 2013).

#### Molecular typing and detection of resistance markers

The genetic relatedness of the *E. aerogenes* isolates were determined by pulsed-field gel electrophoresis (PFGE), as described elsewhere (Kaufmann, 1998). DNA fingerprints were interpreted as recommended by Tenover et al. (1995). The presence of the *bla*_MOX_, *bla*_CMY_, *bla*_LAT_, *bla*_BIL_, *bla*_DHA_, *bla*_ACC_, *bla*_MIR_, *bla*_ACT_, *bla*_FOX_, *bla*_TEM_, *bla*_SHV_, *bla*_CTX-M1, -M2, -M8, -M9, -M25_, *bla*_KPC_, *bla*_GES_, *bla*_IMP_, *bla*_VIM_, *bla*_NDM_, bla_SPM_, bla_GIM_, bla_SIM_, *bla*_OXA-23_, _-48_, _-51_, _-58_, and _-143_ was tested by PCR as previously described (Payne and Thomson, 1998; Poirel et al., 2000, 2011; Perez-Perez and Hanson, 2002; Naas et al., 2008; Higgins et al., 2009; Woodford, 2010; Nordmann et al., 2011).

### Genome sequencing, assembly, and annotation

Genomic DNA was extracted using DNeasy 96 Blood & Tissue Kit (QIAGEN Silicon Valley, Redwood City, USA). DNA quality was assessed using a Bioanalyzer 2100 system (Agilent Technologies, Santa Clara, USA). DNA quantification was performed using Qubit (Thermo Fisher Scientific Inc., Waltham, USA). Illumina sequencing libraries with an average fragment size of 550 bp were prepared using Illumina TruSeq DNA PCR-free LT Kit (Illumina Inc., San Diego, USA). Whole-genome sequencing of paired-end (PE) libraries was performed using a HiSeq 2500 instrument in RAPID run mode (Illumina Inc., San Diego, USA) at the Life Sciences Core Facilities of the State University of Campinas (São Paulo, Brazil). Quality-based trimming and filtering was performed using Trimmomatic version 0.32 (Bolger et al., 2014). PE reads were assembled *de novo* using Velvet version 1.2.10 (Zerbino and Birney, 2008) and contigs were scaffolded using SSPACE version 3.0 (Boetzer et al., 2011). Gene predictions and annotations were performed using NCBI Prokaryotic Genome Automatic Annotation Pipeline (PGAAP; Angiuoli et al., 2008).

### Identification of antibiotic resistance genes

Antibiotic resistance-related genes were predicted using the ResFinder database version 2.1 (Zankari et al., 2012) with the following parameters: “all databases” were used for antimicrobial configuration, type of reads as “assembled genomes/contigs” and thresholds of 98 identity and 80% coverage between sequences. This dataset of resistance genes was complemented with BLASTp searches against the ARDB (Antibiotic Resistance Genes Database) version 1.1 (Liu and Pop, 2009) using “resistance gene complete” database, 40% identity and *e*-value of 0.0001.

## Results

### Resistance profiles

All isolates showed MDR profile and had increased MIC for at least one carbapenem. Information regarding collection date and site, clinical setting, PFGE profile and antimicrobial resistance profiles of each isolate are available in Table 1. Among the four analyzed samples, C10 and D2 were isolated from different body sites of the same patient within a short period of time (a month) and belong to the same PFGE profile. These genomes allow one to analyze the possible genome plasticity between the isolates. D3 and E9 samples were isolated from two patients with an interval of collection date greater than 5 years. D3 and E9 were also interesting because of their sensitivity to meropenem and resistance to ertapenem and imipenem. Surprisingly, E9 showed resistance to carbapenems but not to 3rd (ceftazidime and cefotaxime) and 4th generation (cefepime) cephalosporins (Table 1). All isolates possessed *bla*_AmpC_ and *bla*_TEM_, as detected by PCR. The gene *bla*_CTX-M2_ was found in all isolates except E9. Phenotypic tests (i.e., Modified Hodge test and double-disk synergy) to detect carbapenemases were positive for C10, D2, and E9. However, no class A, B, and D carbapenemase encoding genes were detected by PCR. All isolates tested negative in carbapenem hydrolysis assays.

**Table 1:** **Clinical, phenotypic, molecular data, and genomic features of the four ***Enterobacter aerogenes*** isolates reported in the present work**.

**Sample ID**	***E. aerogenes* C10**	***E. aerogenes* D2**	***E. aerogenes* D3**	***E. aerogenes* E9**
**CLINICAL DATA**				
Date of isolation	09.28.2007	10.12.2007	12.12.2006	01.31.2012
Clinic	Ward	Ward	Ward	ICU^b^
Source	Blood	Catheter tip	BAL^a^	Urine
**MINIMAL INHIBITORY CONCENTRATION (mg/L)**				
Amicacin	**64**	**64**	**64**	**64**
Gentamicin	**>64**	**>64**	**>64**	2
Ceftazidime	**16**	**32**	**16**	0.5
Cefepime	**128**	**>128**	**128**	0.5
Cefotaxime	**>128**	**128**	**128**	0.5
Ertapenem	**32**	**32**	**16**	**2**
Imipenem	**8**	**8**	**32**	**8**
Meropenem	**8**	**8**	2	0.5
Polimyxin	0.25	0.25	0.5	0.25
Ciprofloxacin	**>16**	**>16**	**16**	2
Levofloxacin	**>8**	**8**	**>8**	0.25
Tigecycline	2	2	1	0.5
Doxycycline	**16**	**16**	**64**	8
Minocycline	8	8	8	2
Fosfomycin	**256**	**256**	**>512**	**64**
**MOLECULAR FEATURES**				
PFGE profile	A	A1	B	C
*bla* genes	*bla*_AmpC_, *bla*_TEM_, *bla*_CTX−M2_	*bla*_AmpC_, *bla*_TEM_, *bla*_CTX−M2_	*bla*_AmpC_, *bla*_TEM_, *bla*_CTX−M2_	*bla*_AmpC_, *bla*_TEM_
**GENOMIC FEATURES**				
Estimate genome size (bp)	5,833,521	5,821,782	5,584,745	5,637,471
Genome coverage	208x	182x	137x	197x
Number of scaffolds	58	57	55	59
N50 (bp)	505,999	464,022	505,714	461,836
Number of paired-end reads used	14,346,552	12,939,780	9,406,438	12,891,456
%GC	53.61	53.63	53.69	53.67
Predicted genes	5,636	5,622	5,311	5,402
Predicted protein-coding genes	5,363	5,380	5,067	5,129
tRNAs	82	80	83	85
rRNAs (5S, 16S, 23S)	9, 5, 16	6, 3, 8	8, 4, 9	8, 10, 13
ncRNAs	12	12	13	12
Pseudogenes	149	133	127	145

*Numbers in bold indicate resistance to a given antibiotic*.

a Bronchoalveolar lavage (BAL) and

b *Intensive care unit (ICU)*.

### Genomic features

We obtained between 16,841,714 and 25,138,390 150 bp PE reads per library. After genome assembly, 5,833,521 bp were assembled in 58 scaffolds for C10, 5,821,782 bp were assembled in 57 scaffolds for D2, 5,584,745 bp were assembled in 55 scaffolds for D3 and 5,637,471 bp were assembled in 59 scaffolds for E9. By using the NCBI Prokaryotic Annotation Pipeline, we were able to predict 5,363, 5,380, 5,067, and 5,129 protein-coding sequences in each of the genomes listed above, respectively. Genomic features of the four sequenced genomes are summarized in Table 1.

### Antibiotic resistance genes

A total of 18 enzymes related to antibiotic resistance were identified using ResFinder, ARDB and PGAAP (Table 2). All isolates harbor genes related to: (i) aminoglycoside resistance (genes *aacA4* and *aadA*); (ii) beta-lactam resistance, including genes belonging to class A beta-lactamases (TEM family), class B beta-lactamases (Ribonuclease Z), class C beta-lactamases (CMY/LAT/MOX/ACT/MIR/FOX family) and class D beta-lactamases (OXA-9); (iii) bacitracin resistance (gene *bacA*), and (iv) sulphonamide resistance (gene *sul1*; Table 2). Genes *sul2* and *rmtD* were only identified in *E. aerogenes* D3. The gene *sul2* has been implicated on sulphonamide resistance for inducing high expression levels of the enzyme dihydropteroate synthase (Sköld, 2001), while *rmtD* has been related to aminoglycoside resistance and this variant was identified for the first time in South America in a *P. aeruginosa* isolate in 2005 (Doi et al., 2007). Interestingly, *E. aerogenes* D3 was isolated in 2006, indicating that this variant has spread amongst *Enterobacteriaceae* in Brazil since its first report (Doi et al., 2007).

**Table 2:** **Resistance gene repertoire identified using ResFinder, ARDB, and NCBI annotation pipeline**.

**Protein**	**Reference Sequence**	**C10**	**D2**	**D3**	**E9**
**ENZYMES**					
16S rRNA (adenine(1518)-N(6)/adenine(1519)-N(6))-dimethyltransferase (KsgA)	WP_003829609.1	AW170_18245	AYK88_16575	A1Q75_18030	A1J85_13160
16S rRNA (guanine(1405)-N(7))-methyltransferase RmtD	WP_019726361.1	–	–	A1Q75_26170	–
AacA4 family aminoglycoside N(6')-acetyltransferase (AacA4)	WP_014839929.1 P19650.1	AW170_26985	AYK88_26865 AYK88_26940	A1Q75_26315	A1J85_26740
Aminoglycoside N(3)-acetyltransferase III (AacC3)	P0A255.1	AW170_26910	AYK88_26960	–	–
ANT(3″)-Ia family aminoglycoside nucleotidyltransferase AadA	WP_014325834.1	AW170_26955	AYK88_26945	A1Q75_26045 A1Q75_26310	A1J85_26670 A1J85_26735
Chloramphenicol acetyltransferase III (Cat3)	P00484.1	AW170_27070	AYK88_27075	–	–
Class A beta-lactamase - Beta-lactamase CTX-M-6	O65976.1	AW170_27050	AYK88_27040	A1Q75_26225	–
Class A beta-lactamase - TEM family	WP_010331504.1 WP_000027063.1	AW170_26915 AW170_27230	AYK88_27065 AYK88_27140	A1Q75_26300	A1J85_24665 A1J85_26820
Class A beta-lactamase - TEM family	WP_001398207.1	AW170_26970	–	–	–
Class B beta-lactamase - Ribonuclease Z (metallo-beta-lactamase superfamily)	WP_004890624.1	AW170_13355	AYK88_10035	A1Q75_10920	A1J85_17515
Class C beta-lactamase - CMY/LAT/MOX/ACT/MIR/FOX family	WP_008453751.1	AW170_05580	AYK88_04475	A1Q75_09705	A1J85_14245
Class D beta-lactamase - Beta-lactamase OXA-2	P0A1V8.1	AW170_26980	AYK88_26870	–	–
Class D beta-lactamase - oxacillinase-carbenicillinase (OXA-9)	WP_004153119.1	AW170_26960	AYK88_26950	A1Q75_26305	A1J85_26730
Dihydropteroate synthase type-1 (SulI)	P0C002.1	AW170_27105	AYK88_26880	A1Q75_26340	A1J85_26680
Sulfonamide-resistant dihydropteroate synthase Sul2	WP_001043267.1	–	–	A1Q75_26185	–
Trimethoprim-resistant dihydrofolate reductase DfrA	WP_001611015.1	–	–	A1Q75_26055	A1J85_26660
Undecaprenyl-diphosphatase (BacA)	WP_012907642.1	AW170_01035	AYK88_19635	A1Q75_21735	A1J85_01030
Qnr family quinolone resistance pentapeptide repeat protein	WP_017111199.1	AW170_27090	AYK88_27095	A1Q75_26320	–
**TRANSPORTERS**					
Aminoglycoside/multidrug transporter subunit AcrD	WP_005121895.1	AW170_13975	AYK88_09410	A1Q75_11550	A1J85_16890
Bcr/CflA family multidrug efflux MFS transporter	WP_004202891.1	AW170_03270	AYK88_06790	A1Q75_02780	A1J85_21475
Bcr/CflA family multidrug efflux MFS transporter	WP_008804003.1	AW170_13100	AYK88_10285	A1Q75_10670	A1J85_23465
Chloramphenicol efflux MFS transporter CmlA5	WP_012300772.1	–	–	A1Q75_26050	A1J85_26665
Macrolide ABC transporter permease/ATP-binding protein MacB	WP_004147781.1	AW170_08470	AYK88_01580	A1Q75_06815	A1J85_05705
Macrolide transporter subunit MacA	WP_008805838.1	AW170_08465	AYK88_01585	A1Q75_06820	A1J85_05700
MATE family efflux transporter, multidrug efflux protein	WP_003857645.1	AW170_03255	AYK88_06805	A1Q75_02765	A1J85_21460
Membrane protein, Multidrug resistance efflux pump EmrA	WP_009307711.1	AW170_03350	AYK88_06710	A1Q75_02860	A1J85_21555
MexE family multidrug efflux RND transporter periplasmic adaptor	WP_004121017.1	AW170_01795	AYK88_08270	A1Q75_01550	A1J85_20240
MexE family multidrug efflux RND transporter periplasmic adaptor subunit, multidrug efflux system transporter AcrA	WP_004129915.1 WP_015585499.1	AW170_06375 AW170_10590	AYK88_03675 AYK88_11425	A1Q75_08905 A1Q75_13105	A1J85_15040 A1J85_10280
MexX family efflux pump subunit, multidrug efflux system transporter AcrA	WP_014906857.1	AW170_00035	AYK88_20640	A1Q75_20735	A1J85_00030
Multidrug ABC transporter ATP-binding protein	WP_000422210.1	AW170_13185	AYK88_10200	A1Q75_10755	A1J85_23550
Multidrug efflux RND transporter permease subunit	WP_004901494.1	AW170_10595	AYK88_11430	A1Q75_13110	A1J85_10285
Multidrug efflux RND transporter permease subunit OqxB	WP_015367127.1	AW170_01800	AYK88_08265	A1Q75_01555	A1J85_20245
Multidrug efflux RND transporter permease subunit, multidrug efflux protein AcrB	WP_017899940.1 WP_015571248.1	AW170_00030 AW170_06370	AYK88_20645 AYK88_03680	A1Q75_20730 A1Q75_08910	A1J85_00025 A1J85_15035
Multidrug resistance protein D (EmrD)	WP_008806760.1	AW170_10405	AYK88_11240	A1Q75_12920	A1J85_10095
Multidrug resistance protein MdtB	WP_020244584.1	AW170_12610	AYK88_10840	A1Q75_10110	A1J85_23005
Multidrug resistance protein MdtC	Q7ACM1.1	AW170_12615	AYK88_10845	A1Q75_10105	A1J85_23000
Multidrug resistance protein MdtH	WP_017900739.1	AW170_09790	AYK88_00255	A1Q75_01100	A1J85_19845
Multidrug transporter, multidrug efflux system protein EmrA	WP_009308476.1	AW170_15715	AYK88_14045	A1Q75_16525	A1J85_03195
Outer membrane channel protein TolC	WP_015369648.1	AW170_01095	AYK88_19575	A1Q75_21795	A1J85_01090
Outer membrane component of tripartite multidrug resistance system, putative outer membrane efflux protein MdtP	WP_015369857.1	AW170_16750	AYK88_15075	A1Q75_15485	A1J85_02150
QacE family quaternary ammonium compound efflux SMR transporter	WP_000679416.1	AW170_27110	AYK88_26875	A1Q75_26335	A1J85_26675
Quaternary ammonium compound-resistance protein SugE	WP_001597468.1	AW170_19990	AYK88_18310	A1Q75_19995	A1J85_18375
Tetracycline efflux MFS transporter Tet(D)	WP_001039466.1	–	–	A1Q75_26055	-

Although the four isolates showed carbapenem-resistance, no carbapenemase gene was identified using molecular detection or *in silico* analysis. Hence, it is likely that these isolates employ alternative mechanisms to counter carbapenem effects. Various multidrug efflux transporters were found in the genomes described here (Table 2). They belong to four superfamilies: the major facilitator superfamily (MFS), multidrug and toxic compound extrusion (MATE), ATP-binding cassette (ABC) and resistance-nodulation-cell division (RND). RND type of transporters has been often associated with multidrug resistance of Gram-negative bacteria (Nikaido, 1998). In particular, the RND type genes forming the AcrA-AcrB-TolC efflux pump were found in multiple copies in our isolates (Table 2). Experimental evolution studies of *E. aerogenes* under successive imipenem exposure reported alterations in membrane permeability with complete loss of porins (e.g., Omp35 and Omp36) and overexpression of AcrAB-TolC efflux pumps (Bornet et al., 2003; Thiolas et al., 2005; Lavigne et al., 2012). As a result of efflux pump expression, the *E. aerogenes* isolates showed resistance to carbapenems and other antibiotics, especially fluoroquinolones (Bornet et al., 2003; Thiolas et al., 2005; Lavigne et al., 2012). Given the multiple copies of genes encoding efflux pumps in our isolates, it is possible that an increased expression of AcrAB-TolC efflux pumps could contribute to the observed carbapenem-resistant profiles.

*E. aerogenes* is an emergent nosocomial pathogen with a diversity of mechanisms to circumvent antimicrobial activity. Here we reported the phenotypic screens, genome sequencing, and prediction of putative resistance gene repertoires of four multidrug-resistant *E. aerogenes* isolated between 2006 and 2012. The data reported here may help understand the biochemistry, evolution, and epidemiology of this important pathogen. The material provided in this work may be used in future comparative genomics and molecular epidemiology studies aiming to clarify the resistance profiles and dynamics of multidrug-resistant *Enterobacteriaceae* species.

## Data access

The genome sequence of *E. aerogenes* C10, *E. aerogenes* D2, *E. aerogenes* D3 and *E. aerogenes* E9 have been deposited in DDBJ/EMBL/GenBank under the accession numbers LUTZ00000000, LSOH00000000, LUTT00000000, and LULD00000000, respectively. Data are available in FASTA, annotated GenBank flat file and ASN.1 formats. The respective genome versions described in this paper are LUTZ01000000, LSOH01000000, LUTT01000000, and LULD01000000. Sequencing reads (fastq format) of each isolate were deposited in Sequence Read Archive (SRA) under the accession numbers SRP083774 (*E. aerogenes* C10), SRP083784 (*E. aerogenes* D2), SRP083785 (*E. aerogenes* D3), and SRP083786 (*E. aerogenes* E9). Users can download the data for research purposes, citing the present manuscript as original reference.

## Author contributions

AG, NV, JP, LD, and TV conceived the idea and designed the study. JP performed the sample collections and wet lab experiments. AG and NV carried out the genome analysis. AG, NV, JP, LD, and TV interpreted the data and wrote the manuscript. All authors have read and approved the final version of this manuscript.

### Conflict of interest statement

The authors declare that the research was conducted in the absence of any commercial or financial relationships that could be construed as a potential conflict of interest.
